# Methylenetetrahydrofolate reductase C677T polymorphism and colorectal cancer susceptibility: a meta-analysis

**DOI:** 10.1042/BSR20170917

**Published:** 2017-12-07

**Authors:** Lingyan Xu, Zhiqiang Qin, Feng Wang, Shuhui Si, Lele Li, Peinan Lin, Xiao Han, Xiaomin Cai, Haiwei Yang, Yanhong Gu

**Affiliations:** 1 Department of Oncology, The First Affiliated Hospital of Nanjing Medical University, Nanjing 210029, China; 2 State Key Laboratory of Reproductive Medicine, Department of Urology, The First Affiliated Hospital of Nanjing Medical University, Nanjing 210029, China; 3 Department of Radiation Oncology, The First Affiliated Hospital of Nanjing Medical University, Nanjing 210029, China; 4 Research Division of Clinical Pharmacology, The First Affiliated Hospital of Nanjing Medical University, Nanjing 210029, China

**Keywords:** colorectal cancer, gene polymorphism, MTHFR, meta-analysis

## Abstract

The association between methylenetetrahydrofolate reductase (*MTHFR*) C677T polymorphism and colorectal cancer (CRC) susceptibility has been researched in numerous studies. However, the results of these studies were controversial. Therefore, the objective of this meta-analysis was to offer a more convincible conclusion about such association with more included studies. Eligible studies published till May 1, 2017 were searched from PubMed, Embase, Web of Science, and CNKI database about such association. Pooled odds ratios (ORs) together with 95% confidence intervals (CIs) were calculated to evaluate such association. And the Begg’s funnel plot and Egger’s test were applied to assess the publication bias. This meta-analysis contained 37049 cases and 52444 controls from 87 publications with 91 eligible case–control studies. Because of lack of data for a particular genotype in several studies, all the included studies were analysed barely in the dominant model. Originally, there was no association between *MTHFR* C677T polymorphism and CRC susceptibility (OR =0.99, 95% CI =0.94–1.05). After excluding 13 studies according to their heterogeneity and publication bias, rs1801133 polymorphism was found to reduce the risks of CRC significantly (OR =0.96, 95% CI =0.94–0.99). In the subgroup analysis of ethnicity, there was a significant association in Asians (OR =0.94, 95% CI =0.89–1.00). Furthermore, when stratified by the source of controls and genotyping methods, the positive results were observed in population-based control group (OR =0.97, 95% CI =0.93–1.00) and PCR-restriction fragment length polymorphism (PCR-RFLP) method (OR =0.95, 95% CI =0.91–0.99. The results of the meta-analysis suggested that *MTHFR* C677T polymorphism was associated with CRC susceptibility, especially in Asian population.

## Introduction

Colorectal cancer (CRC) is a critical public health problem, which is the third most commonly diagnosed cancer and the third common cause of cancer deaths in both males and females. There were 134490 new CRC cases and 49190 mortalities by estimation in the United States in 2016 [1]. The colorectal carcinogenesis is a complex multistep progress (a benign adenomatous polyp – an advanced adenoma with high-grade dysplasia – an invasive cancer) with altered expression of oncogenes, tumor suppressor genes and DNA repair genes [2]. However, the etiology of CRC is still unclear. It is known to all that CRC is a multifactorial and multigenic disease, and is influenced by environment conditions, diet habits, genetic mutations, and *Escherichia coli* infection [3,4]. With increasing numbers of studies, more gene polymorphisms were found to contribute to CRC [5]. These single nucleotide polymorphisms (SNPs) can be used as makers for improving cancer diagnosis and determination of treatment plans [6].

As a key enzyme and an important regulator for the metabolism of folate/vitamin B_9_, methylenetetrahydrofolate reductase (*MTHFR*) catalyzes the conversion of 5,10-methylenetetrahydrofolate to 5-methyltetrahydrofolate [7]. Simultaneously, the 5-methyltetrahydrofolate is the main circulatory form of folate in the body and provides a methyl group to convert the amino acid homocysteine into methionine, which is the precursor of S-adenosylmethionine (SAM). SAM is the major methyl donor in the cell and takes part in DNA methylation [8]. Therefore, *MTHFR* not only plays a role in making proteins and other important compounds, but also is an important factor in DNA methylation, synthesis, and repair [9]. The enzyme is encoded by the *MTHFR* gene located on the short arm of chromosome 1-1p36.3 [10]. Previously, several mutations of *MTHFR* gene have been found and *MTHFR* C677T (rs1801133) is the most common type amongst them. *MTHFR* C677T represents an alanine-to-valine substitution at nucleotide position 677 in exon 4 resulting in thermolability and concurrent decreased activity of the enzyme [11,12]. *MTHFR* gene mutations lead to *MTHFR* enzyme dificiency, low plasma folate levels, hyperhomocysteinemia [13,14] and certain diseases such as cardiovascular disease, pregnancy complications, neural defect, and several cancers including CRC [15–21]. With a growing number of studies conducted to explore such association, we hypothesized that rs1801133 was likely to relate to colorectal carcinogenesis.

Many researchers have carried out a large number of studies to examine the potential association between *MTHFR* C677T polymorphism and CRC susceptibility. But, the results are still inconclusive so far. Thus, the aim of this meta-analysis including all available case–control studies was to investigate a more reliable association.

## Materials and methods

We searched several databases including PubMed, Embase, Web of Science, and CNKI database for published studies about exploring the association between *MTHFR* C677T polymorphism and CRC susceptibility till May 1, 2017. The search strategy included listed key words: ‘methylenetetrahydrofolate reductase’, ‘*MTHFR* polymorphism’, ‘C677T’, ‘rs1801133’, and ‘risk or susceptibility’ and ‘colorectal or colon or rectal cancer’. Furthermore, we manually searched the reference lists of clinical trials and former meta-analyses for more relevant studies. When duplicate data appeared in different publications, this meta-analysis only adopted the most recent study or the study with the most complete information. The meta-analysis was on the basis of the preferred reporting items for systematic review and meta-analysis protocols (PRISMA-P) [22]. The eligible studies needed to accord with the following inclusion criteria: (i) case–control studies; (ii) the language was not restricted to English; (iii) investigating the association between *MTHFR* C677T polymorphism and CRC susceptibility; (iv) offering enough raw data to calculate odds ratio (OR) with 95% confidence interval (CI). Additionally, exclusion criteria were as follows: (i) non-case–control studies; (ii) lack of sufficient data for calculating genotype frequency; (iii) case–control studies about examining the relationship between *MTHFR* C677T polymorphism and colorectal adenoma; (iv) duplicated publications.

### Data extraction

In order to guarantee the accuracy of extracted information, two authors individually reviewed each publication and extracted useful data on the basis of the inclusion criteria listed above. When disagreements arose in the course of data extraction, discussion was carried out with other authors until the agreements were reached. The following information were extracted from each study to accomplish a standardized sheet: first author’s name, year of publication, ethnicity of population, source of controls (hospital based or population based), genotyping method, sample size of cases and controls, genotype frequency of rs1801133 in cases and controls, and the results of the Hardy–Weinberg equilibrium (HWE) test.

### Statistical analysis

The relationship between *MTHFR* C677T polymorphism and CRC susceptibility was analyzed by using five models including the dominant model (CT + TT compared with CC), the recessive model (TT compared with CT + CC), the homozygous model (TT compared with CC), the heterozygous model (CT compared with CC), and the allele model (T compared with C). The goodness-of-fit *χ*^2^ test was conducted to evaluate the HWE in control groups and *P*<0.05 was regarded as significant disequilibrium [23]. Stratified analysis were performed by ethnicity, source of controls, and genotyping method. Besides, the pooled OR together with 95% CI were measured to bring out the strength of such association. The fixed effects model (Mantel–Haenszel method) and the random effects model (Dersimonian–Laird method) were selected to use based on heterogeneity in the meta-analysis. If there was no or little heterogeneity, the fixed effects model was used; otherwise, the random effects model was used. Due to only particular genotypes extracted in several studies, the dominant model analysis were carried out for all the included studies [84]. Galbraith graph was performed to explore the impossible cause of heterogeneity [24]. A sensitivity analysis was conducted to assess the stability of the results. Begg’s funnel plot was performed for potential publication bias and Egger’s linear regression test was executed to assess funnel plot asymmetry statistically. If *P*<0.05, publication bias existed [25]. All statistical data analyses were carried out by using Stata software (version 12.0, StataCorp LP, College Station, TX, U.S.A.).

## Results

### Characteristics of the studies

**Figure 1:**
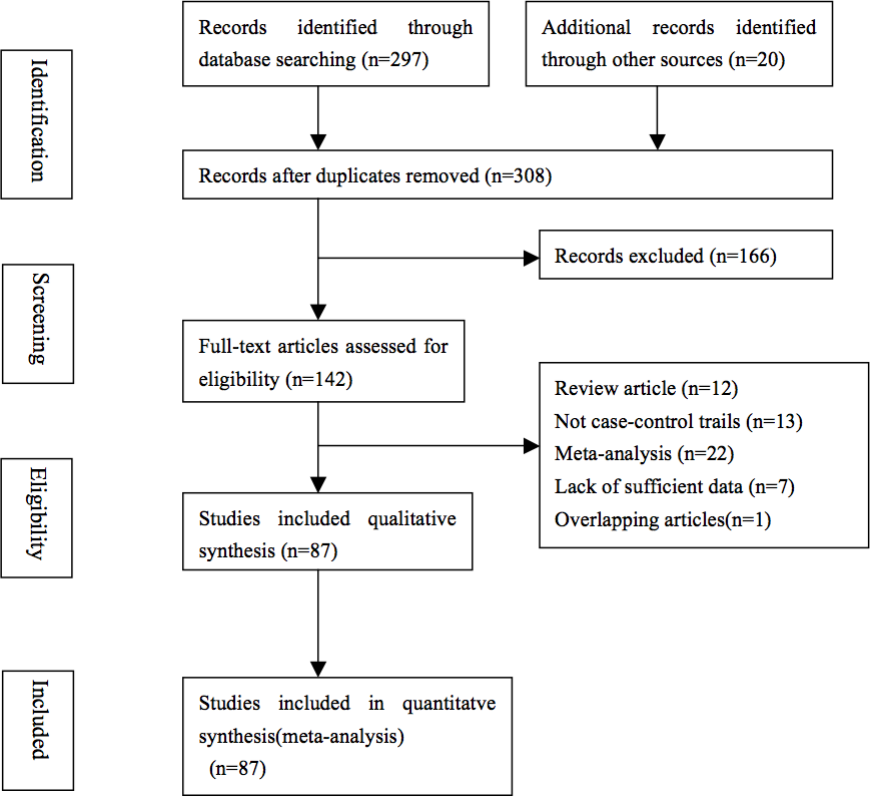
Flowchart of literature search and selection process

According to PRISMA-P, this meta-analysis contained 37049 cases and 52444 controls that were combined from 87 publications with 91 eligible case–control studies to examine the relationship between rs1801133 polymorphism and CRC risks [26–112]. The literature retrieval and selection process are shown in the flowchart in Figure 1. Detailed information of each study were listed in Table 1. The distribution of genotypes in controls was consistent with HWE except 15 studies [33–35,37,39,47,63,71,76,80,87,88,106,110,111]. In these studies, four ethnicities of population were included: Asian, Caucasian, African, and mixed ethnic group. Nine genotyping methods were applied: PCR-restriction fragment length polymorphism (PCR-RFLP), real-time PCR (RT-PCR), PCR-single strand conformation polymorphism (PCR-SSCP), methylation-specific PCR (MS-PCR), mutagenically separated PCR (MSP), MALDI-TOF-MS, Taqman, MassARRAY, and Sequenom. Depending on different sources of control, population-based and hospital-based control groups were distinguished in all the included studies.

**Table 1:** Characteristics of individual studies included in the meta-analysis

MTHFR rs1801133							Case (*n*)			Control (*n*)			
Year	Surname (References)	Ethnicity	SOC	Genotyping	Case	Control	CC	CT	TT	CC	CT	TT	HWE
2016	Haerian [26]	Asian	HB	Taqman	1123	1298	607	421	95	667	523	108	Y
2015	Kim [27]	Asian	PB	PCR-RFLP	477	514	159	248	70	172	265	77	Y
2014	Rai [28]	Asian	PB	PCR-RFLP	155	294	137	17	1	261	31	2	Y
**2014**	**Ozen** [29]	**Caucasian**	**PB**	**RT-PCR**	**86**	**212**	**36**	**32**	**18**	**207**	**5**	**0**	**Y**
**2013**	**Ashmore** [30]	**Caucasian**	**PB**	**RT-PCR**	**625**	**603**	**241**	**309**	**75**	**263**	**259**	**81**	**Y**
**2013**	**Delgado- Plasencia** [31]	**Caucasian**	**HB**	**PCR-RFLP**	**50**	**103**	**32**	**16**	**2**	**44**	**50**	**9**	**Y**
2013	Yousef [32]	Asian	PB	PCR-RFLP	128	116	79	45	4	59	45	12	Y
2012	Lee [33]	Caucasian	PB	Taqman	531	1004	250	229	52	464	391	149	N
2012	Promthet [34]	Asian	HB	PCR-RFLP	112	242	93	18	1	185	49	8	N
2012	Kim [35]	Asian	HB	Taqman	787	656	265	393	129	205	289	162	N
2012	Yin [36]	Asian	HB	RT-PCR	370	370	124	167	79	139	178	53	Y
2011	Sameer [37]	Asian	PB	PCR-RFLP	86	160	59	18	9	121	27	12	N
2011	Vossen [38]	Caucasian	PB	Taqman	1762	1811	737	823	202	795	807	209	Y
2011	Kang [39]	Asian	PB	PCR-RFLP	255	448	87	134	34	145	238	65	N
**2011**	**Zhu** [40]	**Asian**	**PB**	**PCR-RFLP**	**86**	**100**	**29**	**42**	**15**	**49**	**41**	**10**	**Y**
2011	Pardini [41]	Caucasian	HB	PCR-RFLP	666	1376	317	307	42	613	627	136	Y
2011	Kim [42]	Asian	HB	MSP	67	53	30	30	7	15	21	17	Y
**2011**	**Prasad** [43]	**Asian**	**PB**	**PCR-RFLP**	**110**	**241**	**97**	**12**	**1**	**228**	**12**	**1**	**Y**
2011	Li [44]	Asian	PB	PCR-RFLP	137	145	68	54	15	55	64	26	Y
2011	Jokic [45]	Caucasian	PB	Taqman	300	300	139	130	31	142	130	28	Y
2011	Guimaracs(a) [46]	Caucasian	HB	PCR-RFLP	101	188	42	44	15	92	79	17	Y
2011	Guimaracs(b) [46]	African	HB	PCR-RFLP	12	188	6	6	0	92	79	17	Y
**2010**	**Komlosi** [47]	**Caucasian**	**PB**	**PCR-RFLP**	**951**	**939**	**398**	**427**	**126**	**442**	**380**	**117**	**N**
**2010**	**Karpinski** [48]	**Caucasian**	**HB**	**MSP**	**186**	**140**	**74**	**97**	**15**	**71**	**55**	**14**	**Y**
2010	Cui [49]	Asian	PB	PCR-RFLP	1829	1700	622	923	284	540	863	297	Y
2010	Eussen [50]	Caucasian	PB	MALDI-TOF-MS	1329	2366	567	608	154	1019	1076	271	Y
2010	Chandy [51]	Asian	HB	PCR-RFLP	100	86	74	25	1	66	19	1	Y
**2010**	**Naghibalhossaini** [52]	**Asian**	**PB**	**MS-PCR**	**151**	**231**	**64**	**80**	**7**	**150**	**68**	**13**	**Y**
2010	Promthet [53]	Asian	HB	PCR-RFLP	130	130	104	26	0	94	31	5	Y
2010	Yang [54]	Asian	PB	Sequenom	141	165	58	61	22	62	75	28	Y
**2010**	**Fernández - Peralta** [55]	**Caucasian**	**HB**	**PCR-RFLP**	**143**	**103**	**89**	**52**	**2**	**44**	**50**	**9**	**Y**
2010	Zhu [56]	Asian	PB	PCR-RFLP	216	111	88	102	26	50	53	8	Y
2009	Vogel [57]	Caucasian	PB	RT-PCR	689	1793	318	320	51	876	750	167	Y
2009	Iacopetta [58]	Mixed	PB	PCR-SSCP	850	958	382	386	82	428	429	101	Y
2009	Arreola [59]	Caucasian	PB	PCR-RFLP	369	170	124	126	119	59	79	32	Y
2009	Reeves [60]	Caucasian	HB	Taqman	206	211	105	83	18	101	91	19	Y
**2009**	**Awady** [61]	**African**	**HB**	**PCR-RFLP**	**35**	**68**	**6**	**23**	**6**	**44**	**20**	**4**	**Y**
2009	Derwinger [62]	Caucasian	PB	Taqman	544	299	273	216	55	167	107	25	Y
**2008**	**Haghighi** [63]	**Asian**	**HB**	**PCR/pyrosequencing**	**234**	**257**	**117**	**68**	**49**	**94**	**80**	**83**	**N**
2008	Sharp [64]	Caucasian	PB	PCR-RFLP	251	394	117	111	23	170	177	47	Y
2008	Kury [65]	Caucasian	PB	Taqman	1023	1121	435	452	136	457	515	149	Y
2008	Mokarram [66]	Asian	HB	MSP	151	81	64	80	7	40	31	10	Y
2008	Cao [67]	Asian	PB	PCR-RFLP	315	370	109	154	52	121	183	66	Y
2008	Theodoratou [68]	Caucasian	PB	MassARRAY	999	1010	447	441	111	439	455	116	Y
2008	Ekolf [69]	Caucasian	PB	Taqman	220	414	123	85	12	212	160	42	Y
2008	Zhang [70]	Asian	HB	PCR-RFLP	300	299	97	136	67	91	139	69	Y
2008	Guerreiro [71]	Caucasian	HB	Taqman	196	200	94	76	26	84	107	9	N
2007	Osian [72]	Caucasian	HB	PCR-RFLP	69	67	38	25	6	47	17	3	Y
2007	Zeybek [73]	Asian	HB	PCR-RFLP	52	144	18	27	7	64	65	15	Y
2007	Lima(a) [74]	Caucasian	HB	PCR-RFLP	90	300	36	40	14	143	127	30	Y
2007	Lima(b) [74]	African	HB	PCR-RFLP	10	300	4	5	1	143	127	30	Y
2007	Chang [75]	Asian	HB	RT-PCR	195	195	85	86	24	92	87	16	Y
2007	Murtaugh [76]	Mixed	PB	PCR-RFLP	742	970	357	301	84	466	392	112	N
**2007**	**Jin** [77]	**Asian**	**PB**	**Taqman**	**449**	**672**	**182**	**211**	**56**	**211**	**325**	**136**	**Y**
2007	Curtin [78]	Mixed	PB	PCR-RFLP	916	1972	432	402	82	887	858	227	Y
2007	Hubner [79]	Caucasian	PB	Taqman	1685	2691	743	759	183	1173	1192	326	Y
2006	Koushik [80]	Caucasian	PB	Taqman	349	794	166	145	38	355	327	112	N
2006	Battistelli [81]	Caucasian	HB	PCR-RFLP	93	100	32	40	21	30	51	19	Y
2006	Van Guelpen [82]	Caucasian	PB	Taqman	220	415	123	85	12	212	161	42	Y
2006	Wang [83]	Asian	PB	PCR-RFLP	302	291	257	43	2	255	36	0	Y
2006	Chen [84]	Asian	PB	PCR-RFLP	138	340	52	86		133	207		-
2005	Matsuo [85]	Asian	HB	PCR-RFLP	256	771	106	114	36	289	348	134	Y
2005	Landi [86]	Caucasian	HB	RT-PCR	350	309	128	158	64	109	139	61	Y
2005	Marchand [87]	Mixed	PB	PCR-RFLP	817	2021	394	336	87	987	779	255	N
2005	Jiang [88]	Asian	PB	PCR-RFLP	125	339	51	59	15	134	143	62	N
2005	Otani [89]	Asian	HB	MassARRAY	106	222	32	49	25	51	114	57	Y
2005	Miao [90]	Asian	PB	PCR-RFLP	198	420	53	87	58	133	201	86	Y
2004	Kim [91]	Asian	HB	PCR-RFLP	243	225	86	122	35	83	109	33	Y
2004	Ulvik [92]	Caucasian	PB	Taqman	2159	2190	1103	899	157	1092	886	212	Y
2004	Yin [93]	Asian	PB	PCR-RFLP	685	778	270	330	85	278	367	133	Y
2004	Curtin [94]	Mixed	HB	PCR-RFLP	1608	1972	734	724	150	887	858	227	Y
2003	Pufulete [95]	Caucasian	HB	PCR-RFLP	28	76	16	6	6	41	29	6	Y
2003	Plaschke [96]	Caucasian	PB	PCR-RFLP	287	346	133	120	34	149	159	38	Y
2003	Toffoli [97]	Caucasian	PB	PCR-RFLP	276	279	93	145	38	83	140	56	Y
2003	Heijmans [98]	Caucasian	PB	PCR-RFLP	18	793	7	7	4	399	329	65	Y
2003	Huang [99]	Asian	HB	PCR-RFLP	82	82	36	40	6	40	33	9	Y
2003	Barna [100]	Caucasian	PB	PCR-RFLP	101	196	46	48	7	84	97	15	Y
2002	Keku(a) [101]	Caucasian	PB	Taqman/PCR-PFLP	308	539	144	140	24	265	223	51	Y
2002	Keku(b) [101]	African	PB	Taqman/PCR-PFLP	244	329	198	43	3	264	59	6	Y
2002	Marchand(a) [102]	Caucasian	PB	PCR-RFLP	149	171	66	64	19	66	81	24	Y
2002	Marchand(b) [102]	Asian	PB	PCR-RFLP	399	485	170	180	49	191	214	80	Y
2002	Shannon [103]	Caucasian	PB	PCR-SSCP/RFLP	501	1207	249	197	55	533	560	114	Y
2002	Matsuo [104]	Asian	HB	PCR-RFLP	142	241	39	81	22	81	124	36	Y
2002	Sachse [105]	Caucasian	PB	PCR-RFLP	490	592	238	199	53	271	272	49	Y
2002	Chen [106]	Caucasian	PB	PCR-RFLP	202	326	92	92	18	145	132	49	N
**2001**	**Ryan**	**Caucasian**	**PB**	**PCR-RFLP**	**136**	**848**	**49**	**73**	**14**	**439**	**326**	**83**	**Y**
2000	Slattery [108]	Caucasian	PB	PCR-RFLP	232	164	106	107	19	73	71	20	Y
1999	Slattery [109]	Mixed	PB	PCR-RFLP	1467	1821	673	655	139	827	787	207	Y
1999	Park [110]	Asian	PB	PCR-RFLP	200	460	65	107	28	140	246	74	N
1997	Ma [111]	Caucasian	PB	PCR-RFLP	202	326	92	92	18	145	132	49	N
1996	Chen [112]	Caucasian	PB	PCR-RFLP	144	627	67	64	13	280	263	84	Y

These 13 studies in bold were removed afterward because of its heterogeneity and publication bias. Abbreviations: HB: hospital-based control; PB, population-based control; SOC, source of control.

### Results of quantitative synthesis

**Figure 2:**
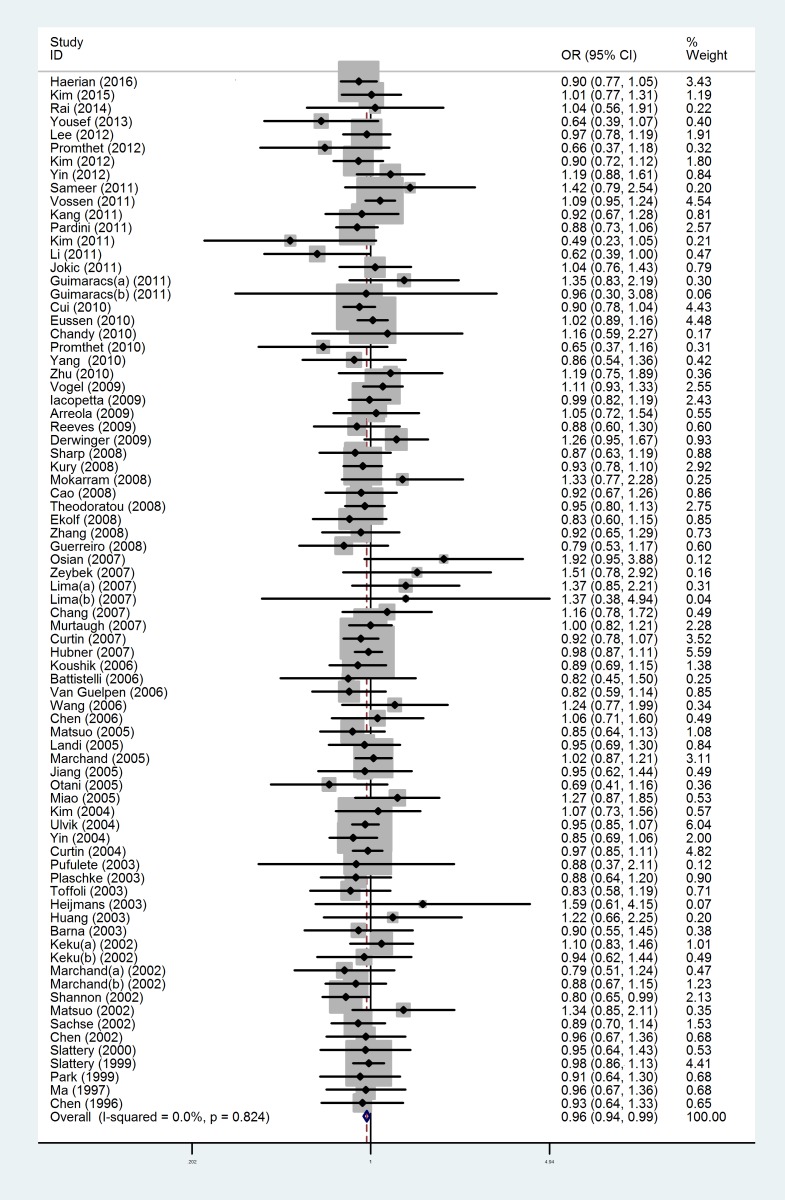
Forest plots of the association between *MTHFR* C677T polymorphism and CRC susceptibility in dominant model after omitting these 13 studies with heterogeneity and publication bias

Initially, there was no association between *MTHFR* C677T polymorphism and CRC susceptibility in the dominant model (OR =0.99, 95% CI =0.94–1.05). 0.94–1.05). Nevertheless, for the sake of looking for possible reasons that might lead to such result, we performed heterogeneity analysis and tested publication bias. According to these results, 13 studies were excluded [29–31,40,43,47,48,52,55,61,63,77,107], the *P*-value was estimated to be 0.824, and the fixed effect model was applied. Ultimately, the results demonstrated that the rs1801133 polymorphism was significantly correlated with the risk of CRC (Figure 2) (dominant model: OR =0.96, 95% CI =0.94–0.99; recessive model: OR =0.90, 95% CI =0.83–0.96; homozygous model: OR =0.88, 95% CI =0.82–0.95; allele model: OR =0.95, 95% CI =0.93–0.98). All detailed results in the present meta-analysis are shown in Table 2.

**Table 2:** Meta-analysis results for the included studies of the association between MTHFR rs1801133 polymorphism and risk of CRC

Variables	Number of studies	Dominant model			Recessive model			Homozygous model			Heterozygous model			Allele model		
		OR (95% CI)	*P*-values	I-squared (%)	OR (95% CI)	*P*-values	I-squared (%)	OR (95% CI)	*P*-values	I-squared (%)	OR (95% CI)	*P*-values	I-squared (%)	OR (95% CI)	*P*-values	I-squared (%)
***rs1801133C>T***		(CT + TT) compared with CC			TT compared with (CT + CC)			TT compared with CC			CT compared with CC			T compared with C		
All	78	**0.96 (0.94–0.99)**	**0.824**	**0.0**	**0.90 (0.83–0.96)**	**<**0.001	**49.9**	**0.88 (0.82–0.95)**	**<**0.001	**42.5**	0.99 (0.96–1.02)	0.950	0.0	**0.95 (0.93–0.98)**	**0.006**	**31.2**
Ethnicity																
Asian	33	**0.94 (0.89–1.00)**	**0.418**	**3.0**	**0.88 (0.77–1.00)**	0.001	**51.2**	**0.86 (0.75–1.00)**	0.001	**49.2**	0.96 (0.91–1.02)	0.933	0.0	**0.94 (0.88–1.00)**	0.002	**47.9**
Caucasian	36	0.97 (0.93–1.01)	0.711	0.0	0.93 (0.83–1.04)	<0.001	57.8	0.91 (0.82–1.01)	0.001	47.7	0.99 (0.95–1.03)	0.505	0.0	**0.96 (0.93–1.00)**	**0.079**	**26.2**
African	3	0.98 (0.67–1.42)	0.866	0.0	0.69 (0.24–2.03)	0.873	0.0	0.72 (0.24–2.15)	0.837	0.0	1.02 (0.69–1.51)	0.852	0.0	0.93 (0.67–1.30)	0.816	0.0
Mixed	6	0.98 (0.92–1.04)	0.959	0.0	**0.83 (0.75–0.92)**	**0.829**	**0.0**	**0.84 (0.75–0.93)**	**0.830**	**0.0**	1.02 (0.95–1.09)	0.967	0.0	**0.95 (0.90–0.99)**	**0.908**	**0.0**
Source of control																
HB	28	0.96 (0.90–1.03)	0.357	7.2	0.97 (0.81–1.16)	<0.001	59.6	0.96 (0.80–1.15)	<0.001	54.4	0.98 (0.92–1.04)	0.550	0.0	0.97 (0.90–1.05)	0.007	44.4
PB	50	**0.97 (0.93–1.00)**	**0.911**	**0.0**	**0.88 (0.81–0.95)**	**0.001**	**43.3**	**0.87 (0.80–0.93)**	**0.012**	**34.1**	0.99 (0.96–1.03)	0.970	0.0	**0.95 (0.92–0.98)**	**0.087**	**22.4**
Geotyping																
Taqman	14	0.96 (0.92–1.01)	0.568	0.0	**0.86 (0.73–1.00)**	**<**0.001	**65.0**	**0.85 (0.74–0.99)**	**0.004**	**57.3**	0.99 (0.94–1.05)	0.460	0.0	**0.94 (0.89–0.99)**	**0.085**	**36.4**
PCR-RFLP	50	**0.95 (0.91–0.99)**	**0.886**	**0.0**	**0.90 (0.81–0.99)**	**0.001**	**43.6**	**0.88 (0.79–0.97)**	**0.005**	**37.5**	0.98 (0.94–1.03)	0.992	0.0	**0.95 (0.91–0.99)**	**0.027**	**30.0**
RT-PCR	4	1.10 (0.97–1.26)	0.746	0.0	1.12 (0.76–1.64)	0.017	70.4	1.15 (0.79–1.66)	0.042	63.4	1.11 (0.96–1.27)	0.771	0.0	1.08 (0.95–1.22)	0.207	34.2

These 13 studies by Ozen et al., Ashmore et al., Delgado-Plasencia et al., Zhu et al., Prasad et al., Komlosi et al., Karpinski et al., Naghibalhossaini et al., Fernández-Peralta et al., Awady et al., Haghighi et al., Jin et al., Ryan et al. were removed [29, 30, 31, 40, 43, 47, 48, 52, 55, 61, 63, 77, 107].

**Figure 3:**
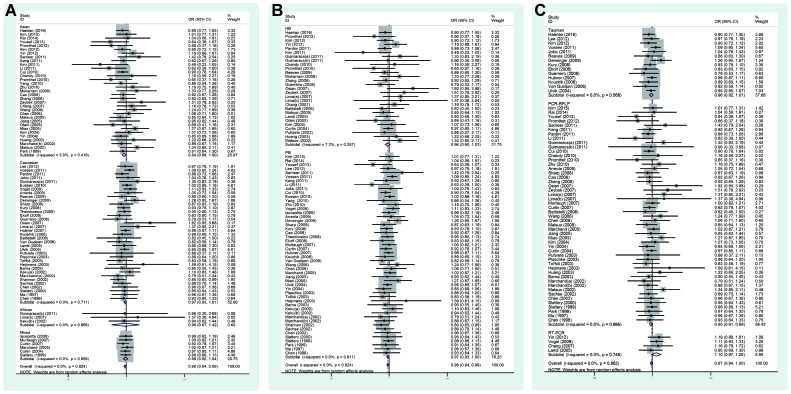
Forest plots of subgroup analysis of the association between *MTHFR* C677T polymorphism and CRC susceptibility in dominant model (**A**) Stratified by ethnicity; (**B**) stratified by source of controls; (**C**) stratified by genotyping method.

In the subgroup analysis of ethnicity, *MTHFR* C677T polymorphism was found to reduce CRC susceptibility in Asians significantly (dominant model: OR =0.94, 95% CI =0.89–1.00 (Figure 3A); recessive model: OR =0.88, 95% CI =0.77–1.00; homozygous model: OR =0.86, 95% CI =0.75–1.00; allele model: OR =0.92, 95% CI =0.88–1.00). Simultaneously, significantly reduced risks were also found in mixed group (recessive model: OR =0.83, 95% CI =0.75–0.92; homozygous model: OR =0.84, 95% CI =0.75–0.93; allele model: OR =0.95, 95% CI =0.90–0.99). Amongst Caucasians, yet significantly reduced risks were only observed in the allele model (OR =0.96, 95% CI =0.93–1.00). Nevertheless, no significant associations were detected in Africans for all genetic models. When stratified by the source of controls, the positive results were observed in population-based control group (dominant model: OR =0.97, 95% CI =0.93–1.00 (Figure 3B); recessive model: OR =0.88, 95% CI =0.81–0.95; homozygous model: OR =0.87, 95% CI =0.80–0.93; allele model: OR =0.95, 95% CI =0.92–0.98). The similar significant associations were absent from hospital-based group for all the genetic models. The stratified analysis by genotyping methods showed that PCR-RFLP method (dominant model: OR =0.95, 95% CI =0.91–0.99 (Figure 3C); recessive model: OR =0.90, 95% CI =0.81–0.99; homozygous model: OR =0.88, 95% CI =0.79–0.97; allele model: OR =0.95, 95% CI =0.91–0.99) and Taqman method (recessive model: OR =0.86, 95% CI =0.73–1.00; homozygous model: OR =0.85, 95% CI =0.74–0.99; allele model: OR =0.94, 95% CI =0.89–0.99) were significantly correlated with risks of decreased CRC. However, RT-PCR method was not relevant to significant associations for all genetic models. In conclusion, the present meta-analysis suggested that *MTHFR* C677T polymorphism was connected with CRC susceptibility.

### Test of heterogeneity

**Figure 4:**
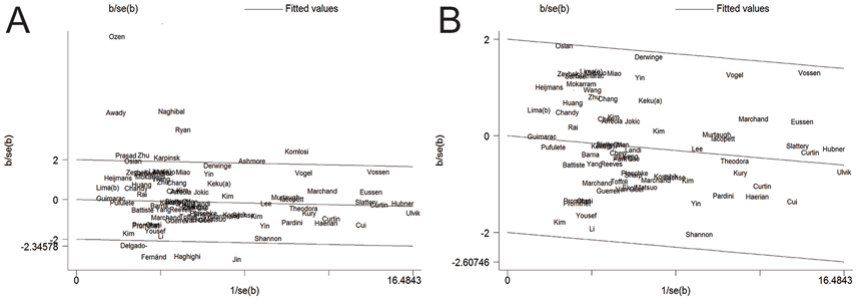
Galbraith plot of the association between *MTHFR* C677T polymorphism and CRC susceptibility in dominant model (**A**) Before removing these 13 studies. (**B**) After the exclusion of these studies.

Heterogeneity analysis was performed in this meta-analysis, and heterogeneity was significantly observed between all the included studies in the dominant model (*I*^2^ =62.0%, *P*<0.001; Figure 4A). In addition, the Galbraith radial plot illustrated heterogeneity obviously. Meanwhile, it specifically pointed out 13 studies that might have led to the obvious heterogeneity and insignificant results of the meta-analysis [27–29,38,41,45,46,50,53,59,61,75,105]. After excluding 13 studies, the heterogeneity decreased significantly (*I*^2^ =0.0%, *P*=0.789; Figure 4B) in the present meta-analysis.

### Publication bias

**Figure 5:**
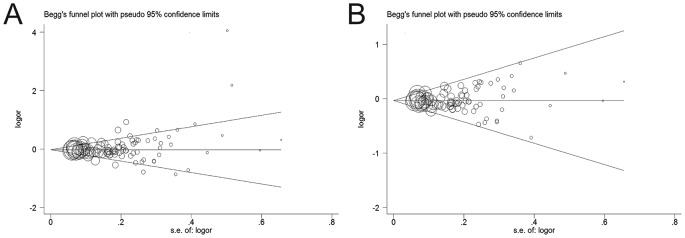
Begg’s funnel plot of publication bias test (**A**) Before omitting these 13 studies. (**B**) After the exclusion of these studies.

The Begg’s funnel plot and Egger’s test were performed to assess the publication bias. Initially, the Begg’s funnel plot was asymmetrical obviously with all the included studies and it suggested a potential publication bias (Begg’s test: *P*=0.103; Egger’s test: *P*=0.058; Figure 5A). After the removal of 13 studies mentioned above [27–29,38,41,45,46,50,53,59,61,75,105], the plots seemed to have a symmetrical distribution in the funnel plot and then Egger’s test was used to provide statistical evidence (Begg’s test: *P*=0.369; Egger’s test: *P*=0.136; Figure 5B). No significant publication bias was observed in the present studies.

### Sensitivity analysis

In order to distinguish the impact of each study on the pooled ORs, we conducted one-way sensitivity analysis. Each time one study was omitted, meta-analysis was repeated and the statistical significance of the results was not changed. Therefore, the results confirmed that the present meta-analysis was relatively stable and reliable.

## Discussion

*MTHFR* is a key enzyme in the folate metabolism and may play a role in the CRC carcinogenesis. It is an essential enzyme in the catalytic reaction that converts 5,10-methylenetetrahydrofolate into 5-methyltetrahydrofolate. On one hand, 5,10-methylenetetrahydrofolate takes part in the thymidylate synthesis. On the other hand, 5-methyltetrahydrofolate promotes methionine synthesis and SAM-mediated methylations. In brief, *MTHFR* has an influence on DNA synthesis, methylation, and repair [113]. The *MTHFR* polymorphisms result in the decreased enzyme activity and then low levels of plasma folate and high homocysteine come to light. Folate is one of water-soluble B vitamins that takes part in various biochemical reactions with its activity to provide or accept one-carbon units [13]. Folate deficiency is likely to contribute to the development of CRC, and several mechanisms may explain how it leads to CRC, including DNA strand breaks, abnormal DNA methylation, and impaired DNA repair [114].

Several polymorphisms have been reported about the *MTHFR* gene coding relevant enzyme, and *MTHFR* C677T polymorphism is the most common one. Heretofore, various studies conducted to detect such association and obtained inconsistent results. Chen et al. [112], first reported that *MTHFR* variant homozygous (TT) genotype was closely linked to reduced incidence of CRC with low consumption of alcohol. In the next few years, similar results were replicated by several other studies [109–111]. However, another study of a homogeneous northern European population obtained different conclusions that *MTHFR* CT heterozygote had a significantly increased risk of developing CRC and no increased cancer risk was observed in TT homozygotes [107]. In addition, a hospital-based case–control study conducted by Matsuo et al. [104] found no significant relativity between *MTHFR* C677T and the risks of CRC. Owing to the difference in study design and the sample size, the different ethnicity, and the diverse stratification, these controversial results were found in published studies. Hence, meta-analysis is essential to be carried out by combining all studies that meet the requirements to get more precise conclusions.

In recent years, there were several meta-analyses performed to elucidate the association of *MTHFR* C677T polymorphism and the susceptibility to CRC before [26,115–118]. Compared with them, this meta-analysis included the most eligible reported studies with the largest sample size and had no restrictions in ethnicity. Since the quality of included documents were disequilibrium, our initial analysis achieved no significant results with all eligible studies. In order to obtain more reliable results, the final conclusion were obtained excluding 13 studies in accordance with the analysis of heterogeneity and publication bias. In this meta-analysis, the pooled conclusions revealed that rs1801133 polymorphism significantly reduced the risk of CRC in the dominant model. The findings agreed with the overwhelming majority results reported by the published studies.

When stratified by ethnicity, there was a significant association with reduced risks of CRC in Asians. The result was consistent with the two previous meta-analysis based on the Asians [116,117]. Zhong et al. [118], carried out a meta-analysis obtaining similar results in East Asians and further subgroup analyses by country identified such association in Korea and Japan. Nevertheless, the recent meta-analysis failed to identify that rs1801133 polymorphism was connected with CRC susceptibility in Iranian population [26]. By means of stratified analysis based on the source of controls and genotyping methods, the positive results were observed in population-based control group and PCR-RFLP method. In general, the source of controls included healthy individuals and patients without CRC. Since the risks of CRC varies amongst individuals over a few years, it might have an impact on the results of relevant studies and make them unreliable. Therefore, inclusion criteria should be improved and studies with large sample sizes should be accepted. In the subgroup of genotyping method, there were nine methods applied for genotyping such as PCR-RFLP, RT-PCR, PCR-SSCP, MS-PCR, MSP, MALDI-TOF-MS, Taqman, MassARRAY, and Sequenom in the including studies. Specific methods and steps were described in each article. Amongst these 87 studies, the majority method was PCR-RFLP. Different methods have their own merits, and when all included studies used the same method, the final results would be more reliable.

In the present meta-analysis, we had obtained weak associations significantly with a large sample size. However, the potential limitations of the meta-analysis should be acknowledged. First, this meta-analysis was based on unadjusted effect estimates and 95% CI, and the influence of multiple cofactors such as age, gender, diet habits including intake of alcohol and consumption of cigarette, the level of folate, and the other environmental factors should be taken into consideration. Second, because of incomplete data of some genotypes, only the dominant model was analyzed in all the included studies. Third, we did not perform stratification analysis by serum folate levels, locations of the tumor and so on, which might result in confounding bias. In addition, after excluding 13 studies according to the analysis of heterogeneity and publication bias, the heterogeneity decreased significantly and the publication bias seemed to disappear. However, the selection bias existed because all the studies were published. Furthermore, the gene–gene and gene–environment interactions were not mentioned in this meta-analysis. In addition, the potential roles of the gene polymorphism which were hidden or magnified by other interactions were omitted.

## Conclusion

In summary, the present meta-analysis revealed that there was a significant association between *MTHFR* C677T polymorphism and susceptibility to CRC. Simultaneously, the TT genotype of *MTHFR* C677T polymorphism could reduce the risk of CRC. In addition, the associated risk of CRC was also reduced in Asians and those studies with population-based controls and used the PCR-RFLP method. Therefore, detection of the *MTHFR* C677T polymorphism might be used as markers for CRC prediction and treatment selection.

## Abbreviations

CI: confidence interval
CRC: colorectal cancer
HWE: Hardy–Weinberg equilibrium
MSP: mutagenically separated PCR
MS-PCR: methylation-specific PCR
MTHFR: methylenetetrahydrofolate reductase
OR: odds ratio
PCR-RFLP: PCR-restriction fragment length polymorphism
PCR-SSCP: PCR-single strand conformation polymorphism
PRISMA-P: preferred reporting items for systematic review and meta-analysis protocol
RT-PCR: real-time PCR
SAM: S-adenosylmethionine
